# Silicon balance in human volunteers; a pilot study to establish the variance in silicon excretion versus intake

**DOI:** 10.1186/1743-7075-11-4

**Published:** 2014-01-09

**Authors:** Supannee Pruksa, Atitaya Siripinyanond, Jonathan J Powell, Ravin Jugdaohsingh

**Affiliations:** 1 Faculty of Science and Technology, Loei Rajabhat University, Loei-Chiangkan Road, A. Muang, Loei 42000, Thailand; 2 Applied Analytical and Inorganic Chemistry Program, Department of Chemistry, Faculty of Science, Mahidol University, Rajthevee, Bangkok 10400, Thailand; 3 MRC Human Nutrition Research, Elsie Widdowson Laboratory, Fulbourn Road, Cambridge CB1 9NL, UK

**Keywords:** Silicon, Orthosilicic acid, Absorption, Balance studies, Urine, Faeces

## Abstract

**Background:**

Accumulating evidence suggests a role for silicon in optimal connective tissue health. Further proof of its importance/essentiality may be provided by studies involving imposed depletion followed by ^29^Si challenge to estimate metabolic balance. Prior to conducting these expensive studies, we first established the variance of estimating normal Si excretion *versus* intake using a single oral dose of typical dietary Si, orthosilicic acid.

**Methods:**

Healthy volunteers were recruited from Loei Rajabhat University, separated into two matched groups (three males and three females/group) and maintained on a standardized diet for the three study days. One group ingested 500 ml water containing orthosilicic acid (28.9 mg Si) and the other group received 500 ml water alone, all on a fasted stomach. Blood samples and total urine and faeces were collected over the 48 h post-dose period and 24 h before-hand (baseline) and analysed for silicon by inductively coupled plasma optical emission spectrometry.

**Results:**

Serum Si analysis confirmed the ready absorption of silicon from the orthosilicic acid solution. Mean total urinary and faecal Si excretions over the 24 h post-dose period accounted for 57 ± 9.5% and 39 ± 9.4% of the ingested dose, respectively. Thus in total 96.3 ± 5.8% of the ingested dose was recovered in faecal plus urinary excretions over the 24 h post-dose period.

**Conclusions:**

We report that in healthy subjects (presumably in Si balance), the ingestion of a soluble dose of dietary Si results in the same quantity (within analytical error) being excreted within 24 h. It is currently not known if this all originated from the dose solution or if there was some exchange with the body Si pool but, given the low variance in these silicon balance data, isotopic studies are now merited.

## Background

Silicon is a critical element in the biology and/or survival of a number of lower life forms, including diatoms, certain sponges and many plants [1-4]. In humans and other mammals its role (if any) is less well defined despite being a common dietary trace element (20–50 mg/day is ingested by adults in western populations [5-8]). Indeed, the environmental ubiquity and limited (bio) chemistry of silicon have led to claims that its ingestion, ready absorption and excretion by mammals are all simply inevitable consequences of oral exposure to a small soluble molecule (orthosilicic acid, Si(OH)_4_) that ‘washes through’ the system and has no biological function [9,10]. Against this, evidence is accumulating to suggest that, in mammals, silicon plays an important role in optimal connective tissue health [11-14]. Its exact role/function remains unestablished, but there is evidence to suggest it’s involved in the synthesis and/or stabilisation of extracellular matrix components, namely collagen, and in the proliferation of connective tissue cells [9,14]. There is also evidence to suggest that silicon is carefully conserved when dietary deficiency is imposed [15,16]. To translate these findings to humans, and provide more evidence for its essentiality, balance studies using Si isotope(s) following a low silicon diet may demonstrate (i) retention of silicon following ingestion and (ii) whether ingested and absorbed silicon displaces some endogenous silicon or is simply washed through.

Despite the simple form of balance studies, where quantitative faecal and urinary excretion of a substance (and/or metabolites) are compared to the amount of substance ingested, they are fraught with difficulties [17]. Faecal collection and analysis is especially demanding: gastrointestinal transit times vary between individuals and temporary mucosal retention of a substance may also occur, adding further variability. Volunteers must provide complete collections and analysis of different fractions and sample types causes inevitable compound error.

For silicon, a basic human balance study, no matter how precise or accurate, would tell us little about the homeostasis of the element. If silicic acid is not utilised/metabolised, but is washed through the system, then a perfect study would recover 100% of the ingested dose. On the other hand, if it were utilised and/or metabolised, a 100% recovery would *still* be expected assuming that volunteers are themselves in balance (i.e. not deficient). Nonetheless, the value of such a study would be to determine what sort of variance one might expect in balance if this were to be attempted in subsequent isotope and/or depletion studies (i.e. are these expensive and time consuming studies worth doing?). It would also have the added value of confirming whether urinary silicon, which is typically used to estimate silicon absorption in humans, is a valid measure for this purpose.

Here we sought to determine the balance in excretion of silicon (faecal and urinary) *versus* intake, using a single oral dose of silicic acid (28.9 mg Si) in human volunteers on a standardized diet.

### Subjects and methods

#### *Subjects*

Fourteen healthy volunteers (seven males and seven females, aged 18–23 years old) were recruited by advertisement on notice boards at Loei Rajabhat University, Thailand. Two subjects (one male and one female) were excluded due to fainting during blood collection at the screening stage. The remaining 12 subjects were self-reportedly healthy with normal renal function, as assessed by serum creatinine, and were not taking Si supplements and/or medicines containing Si and were not pregnant or lactating. The 12 subjects were divided into two groups of six, matched for age, body mass index (BMI) and male to female ratio. One group ingested 500 mL UHP water (Control group) and the other group 500 ml of the Si supplement solution (28.9 mg Si; Si-supplemented group). Anthropogenic data (age, height, weight, BMI and serum creatinine) were collected for each participant and there was no significant difference in subject characteristics between the two groups (Table 1). The study was conducted according to the guidelines laid down in the Declaration of Helsinki and was approved by the Loei Rajabhat University Local Research Ethics Committee. All participants gave signed written consent following oral and written explanation of the study details.

**Table 1:** Characteristics of the study volunteers

**Characteristics**	**Control group (3 M & 3 F)**		**Si-supplemented group (3 M & 3 F)**	
	**Mean ± SD**	**Range**	**Mean ± SD**	**Range**
Age (y)	21.2 ± 1.9	(18.1–23.1)	21.0 ± 1.3	(19.0–22.0)
Weight (kg)	55.1 ± 7.3	(45.2–65.4)	56.4 ± 6.6	(50.0–66.8)
Height (cm)	163 ± 10	(155–176)	165 ± 8	(157–176)
BMI (kg/m^2^)	20.6 ± 1.7	(18.1–22.7)	20.8 ± 2.5	(18.2–25.0)
Serum creatinine^*1*^ (mg/dL)	0.92 ± 0.15	(0.80–1.10)	0.88 ± 0.15	(0.80–1.10)
Baseline 24 h Si excretion:				
Urine (mg/24 h)	12.48 ± 2.26	(9.72–15.31)	12.93 ± 2.85	(9.11–16.34)
Faeces (mg/24 h)	9.50 ± 1.41	(7.99–11.48)	9.35 ± 2.01	(6.68–11.47)

^*1*^Normal creatinine levels: male 0.9-1.3, female 0.6-1.1 mg/dL.

### Materials

Glassware was avoided throughout the study to prevent Si contamination. Ultra high purity (UHP) water was from a water purifier (Labscan Asia Co Limited, Bangkok, Thailand). The stock basic sodium silicate solution was from Lakehead University, Canada (Professor Stephen Kinrade). The stock silicon ICP standard solution (1,000 mg/L Si) was from Merck Ltd (Poole, UK). Nitric acid (65% (w/v) HNO_3_) and hydrochloric acid (37%) were high purity from RCI Labscan Limited (Bangkok, Thailand). Polypropylene tubes (15 and 50 mL) were from Elkay Laboratory Products UK Ltd (Basingstoke, UK). Polypropylene bottles (30 and 2,000 mL) were from VWR International (Poole, UK). All intravenous catheters (1.2 × 45 mm) and plastic syringes were from Nipro Ltd (Pranakhonsriayuthaya, Thailand). Pasteur pipettes (3.5 mL), used for sample transfer, were from Greiner Bio-One Limited (Stonehouse, UK). Pipette tips (100–1,000 μL) were from Hycon (Biomed C. Ltd., Bangkok, Thailand).

#### Preparation of Si supplement

The Si supplement (orthosilicic acid solution, OSA) was prepared fresh, just prior to ingestion, by dilution of the stock basic sodium silicate solution (1.58 mol Si/L or 45.72 g Si/L) into UHP water and pH neutralization to 7.2 with HCl. The Si concentration in the test solution (2.06 mmol/L or 57.78 mg/L) was confirmed by inductively coupled plasma – optical emission spectrometry (ICP-OES; Perkin Optima, model 2100 DV).

#### Study design

**Figure 1:**
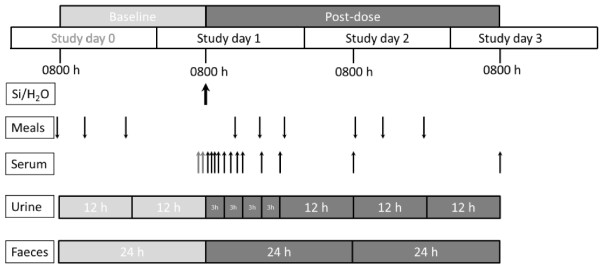
**Study design. Summary of the timing of ingestion of the test solutions (Si-supplement or ultra-high purity water; Si/H**_**2**_**O shown by *thick black arrow*) and standardised meals (*down arrows*), and the collection of serum (*thin up arrows*), urine and faecal samples (collection periods indicated).** Pre-dose (baseline; *light grey*) and post-dose (*dark grey*) collections are indicated.

Twenty four hours prior to ingestion of the test solutions (study day 0), all subjects collected 24 h urines (as two 12 h collections) and faeces (one 24 h collection) for baseline Si measurements. All subjects fasted overnight from 22.00 h and reported, still fasted, to Loei Rajabhat University the following morning (study day 1) at 08.00 h where they emptied their bladder and bowel into containers (part of 24 h baseline collections) and had an intravenous catheter inserted into a forearm vein. Two 10 mL blood samples were collected into polypropylene tubes for baseline Si measurements. Subjects were given 500 mL of the test solution (Si supplement or water) and asked to consume it as quickly as possible, i.e. within 10–15 min. Thereafter, further blood samples (10 mL) were collected at 30 min intervals for the first 2 h, 1 h intervals for next 4 h and finally at 9, 12, 24 and 48 h post-ingestion (the latter two were collected at 08.00 h on study days 2 and 3). Subjects also collected their urine as four 3 h collections (i.e. 08.00-11.00 h, 11.00-14.00 h, 14.00-17.00 h and 17.00-20.00 h) plus three 12 h collections (i.e. 20.00 h study day 1 to 08.00 h study day 2, 08.00 h study day 2 to 20.00 h study day 2, 20.00 h study day 2 to 08.00 h study day 3) in separate pre-weighed, pre-cleaned plastic containers. Additionally, subjects also collected all their faeces over the 48 h post-dose period as two 24 h collections (08.00 h study day 1 to 08.00 h study day 2, 08.00 h study day 2 to 08.00 h study day 3) in containers provided. A summary of the study design is shown in Figure 1.

#### Standardized meals

All 12 participants received the same meals, and the same amount of each meal, for breakfast, lunch and dinner during the study/sample collection periods: namely, study day 0 (baseline collection), study day 1 and study day 2. Males and females received the same amount of each meal and therefore their silicon intakes were identical. The contribution of Si from the three meals was estimated from reference food Si values [5,18] or from direct analysis (i.e. drinking water) and was, on average, 24 mg Si/day over the study period (Table 2). Meals were eaten as follows: breakfast at 08.00 h for study day 0 and study day 2, and at 12.30 h for study day 1 as subjects remained fasted until then; lunch at 12.30 h for study day 0 and study day 2, and at 16.30 h on study day 1; dinner at 18.30 h for study day 0 and study day 2, and at 20.30 h on study day 1. The total intake of Si from the meals on study day 1 was 23.86 mg. All subjects ate at the same times.

**Table 2:** **Estimated silicon content**^***1 ***^**of the meals (mg Si/meal) provided to all participants during the study period**

**Meals**	**Components**	**Day 0**^***3 ***^**(mg Si)**	**Day 1 (mg Si)**	**Day 2 (mg Si)**
Breakfast	Egg fried rice with pork/chicken, orange juice, drinking water^*2*^	6.78	7.70	7.34
Lunch	Rice with chicken, drinking water^*2*^, yogurt, sponge cake	7.59	7.33	9.01
Dinner	Rice with stir-fried pork/chicken with kale, melon, drinking water^*2*^	9.93	7.43	7.97
	Total Si intake per day	24.29	23.86	23.91

^*1*^Calculated from reference food Si values (5, 18).

^*2*^Produced from tap water by reverse osmosis (Si concentration = 0.67 mg Si/L as analysed here by ICP-OES).

^*3*^Day 0 = pre-dose, baseline collection period, conducted 24 h prior to ingestion of the test solutions on day 1.

#### Sample preparations

##### Serum samples

Blood samples were collected in 15 mL polypropylene tubes and left to stand for at least 1 h at room temperature to clot. The clotted blood samples were then centrifuged (Hettich Zentrifugen, Tuttlingen, Germany) at 3,000 rpm for 10 min at room temperature. The separated serum fractions were collected into new 15 mL polypropylene transport tubes and stored at -20°C until elemental analysis. Prior to analysis, the serum samples were thawed at room temperature (23°C) and then diluted 1 + 4 with 0.25% (v/v) high purity HNO_3_.

##### Urine samples

Urine collections were weighed and volumes calculated assuming a density of 1. After thorough mixing, a 10 mL homogenous sample was collected into a 30 mL polypropylene bottle and diluted with equi-volume 0.7% (v/v) high purity HNO_3_ (i.e. 1 + 1 dilution) to reduce any precipitation during storage [19]. The diluted samples were stored at 4°C until elemental analysis. Prior to analysis, the diluted samples were incubated overnight in their closed containers at 40°C in an oven to dissolve any precipitates that may have formed during storage [19]. Samples were cooled to room temperature prior to total elemental analysis for Si.

##### Faecal samples

Faecal collections were weighed and after thorough manual mixing with a disposable wooden spatula, a homogenous sample (from each collection) was collected into a 50 mL polypropylene bottle and stored at -20°C. Prior to analysis, approximately 0.25-0.5 g of the faecal samples was digested with an equi-volume (5 mL) of concentrated (65% (w/v)) HNO_3_ and hydrogen peroxide (30-40%) at room temperature for 24 h. These were incubated at 40°C until total digestion was obtained. Sample (acid) blanks were similarly prepared. An aliquot (1 mL) of the digested samples and sample blanks were diluted with 5 mL UHP water prior to total element analysis for Si.

#### Total elemental analysis

Total elemental analysis for Si was carried out (at 251.611 nm) by ICP-OES; Perkin Elmer Optima model 2100 DV, equipped with a Cross flow nebuliser and Cyclonic spray chamber. Nebulizer flow rate was 0.8 L/min. Peak area was 7.7 points and integration times were 20 seconds/analysis/element. Sample flow rate was 2 mL/min. Matrix matched standards, sample blanks, diluents and quality control samples were run alongside the samples.

##### Serum, urine and faecal samples

The diluted serum, urine, and faecal samples from the same subject were analyzed together in the same batch. Sample-based standards were prepared in the pooled diluted sera, baseline urine, or baseline faecal samples using the 1,000 mg/L Si ICP standard solution.

##### Test solutions

The Si supplement solution, UHP water and drinking water (part of standardized meals) were also analyzed for Si by ICP-OES using appropriate standards.

##### Sample diluents

As the serum and urine samples were diluted with 0.25-0.7% HNO_3_, Si content of the acid diluents was also measured by ICP-OES. Although minor, this contaminant Si was subtracted from each of the sample data.

#### Statistical analysis

Sample size (power) calculation was based on the available data on urinary Si excretion [20]. No previous data exist for faecal Si excretion. A relative standard deviation (σ) of 9.4% [20] was estimated for the variance in urinary Si and a potential difference of 20% for the excretion of urinary Si between the Si supplement and water test solutions was assumed, with 90% power at a 5% significance level. Sample size formula for the difference of two independent means was used for the calculation and six completed subjects were the minimum required for each test solution.

Area under the curve (AUC) of serum Si was calculated using the linear trapezoidal rule [21]. Due to a small number of subjects in each group, differences in serum AUC, and in urinary and faecal excretions of Si, between the two groups (Si vs. control), were analysed non-parametrically using the Mann–Whitney Rank test. Statistical analyses were two sided and a *P* value ≤ 0.05 was considered significant. SPSS for Windows version 13.0 (SPSS Inc., Chicago, Illinois, USA) was used for all statistical analyses.

## Results

Gender specific analysis showed no significant difference in silicon absorption, excretion (urinary and fecal) and balance between male and female subjects and so the combined (pooled) dataset is shown for clarity and because of the small number of subjects.

### Serum Si absorption

**Figure 2:**
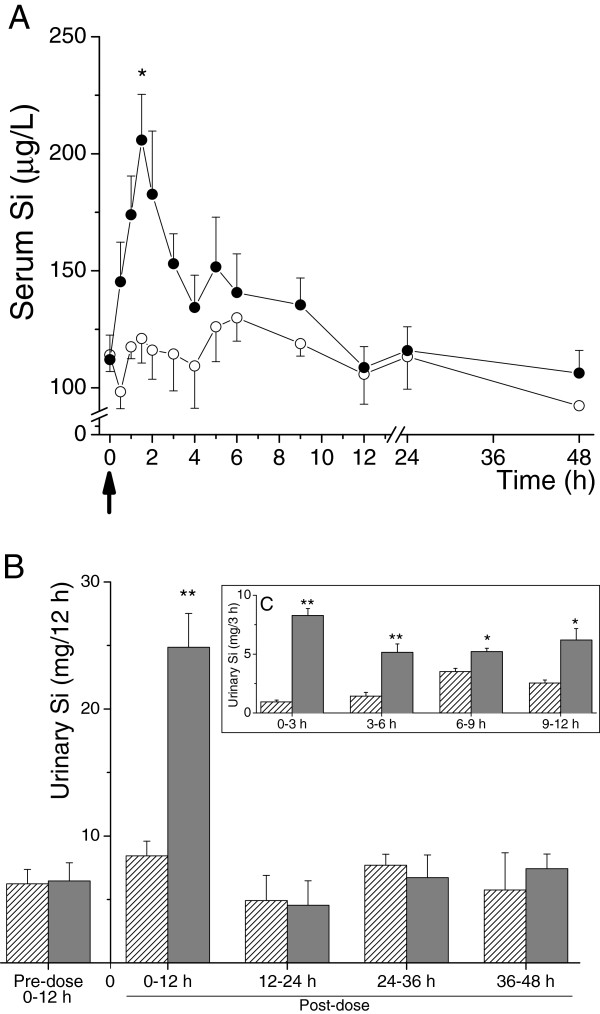
**Serum Si absorption and urinary Si excretion following ingestion of the test solutions. (A)** Serum silicon absorption. Serum Si following ingestion of UHP water alone (◯―◯; Control group) or following ingestion of UHP water containing 28.9 mg Si as orthosilicic acid (●―● Si-supplemented group). Data are means ± SE of 6 subjects per group. The large arrow below the x-axis indicates the time of ingestion of the test solutions (i.e. t = 0). *P = 0.009 (Mann–Whitney Rank test). **(B and C)** Urinary silicon excretion. Urinary excretion of Si following ingestion of UHP water alone (*stripped bars*; Control group) and UHP water containing 28.9 mg Si as orthosilicic acid (*grey bars*; Si-supplemented group). **(B)** Silicon levels in the 12-h urine collections over the 48 h study period. **(C)** The *insert* shows more detailed analysis of Si output over the first 12 h following ingestion of the test solutions. Data are means ± SD of 6 subjects per group. *P = 0.009, **P = 0.002 (Mann–Whitney Rank test).

Mean baseline serum Si concentrations were similar in the control and the Si-supplemented groups (114 ± 17 μg/L (range 85 – 130 μg/L) vs. 112 ± 26 μg/L (range 83 – 148 μg/L)) and remained close to baseline in the control group following ingestion of UHP water (Figure 2A). In contrast, and as expected, serum Si concentrations increased markedly above baseline following ingestion of the orthosilicic acid solution (28.9 mg Si in UHP water) in the Si-supplemented group, with peak Si concentration (206 ± 48 μg/L; range: 133 – 250 μg/L) observed 1 to 2 hours post-ingestion (Figure 2A). Thereafter, serum Si concentrations began to drop rapidly back towards baseline concentrations. However, the ingestion of meals at 4.5 h and 8.5 h post-dose maintained serum Si concentrations above baseline in the Si-supplemented group and increased Si concentration above baseline in the control group, until 12 h post-dose. Area under the curve (AUC) of serum Si over the 24 h period was not significantly different between the two test solutions. However, AUC for the period prior to breakfast, i.e. 0–4 h post-dose, was significantly higher following ingestion of orthosilicic acid solution, compared to UHP water alone (648 ± 137 mg h/L (range 459 – 819 mg h/L) vs. 453 ± 104 mg h/L (range 319 – 630 mg h/L); *P* = 0.04).

### Urinary Si excretion

Mean total baseline 24 h urinary Si excretion was 12.48 ± 2.26 (range 9.72 – 15.31) and 12.93 ± 2.85 (range 9.11 – 16.34) mg, respectively, in the control and Si-supplemented groups and did not change markedly in the control group following ingestion of UHP water (13.34 ± 2.48 mg; range 11.36 – 18.21 mg). In contrast, in the Si-supplemented group, ingestion of the orthosilicic acid solution (28.9 mg Si in UHP water) led to a marked increase in urinary excretion of Si in the 0–12 h post-dose collection (*P* = 0.002; Figure 2B). A more detailed analysis of the 0–12 h collection in the Si-supplemented group showed that the peak increase in Si output was at 0–3 h post-dose (Figure 2C). Again, the ingestion of meals at 4.5 h and 8.5 h post-dose maintained urinary Si output above baseline in the 6–9 h and 9–12 h post-dose collections, as clearly mirrored in the control group (Figure 1C). Urinary Si output in the remaining collections (12–24 h, 24–36 h and 36–48 h), were comparable to baseline levels and similar between the two groups.

The increase in urinary Si output in the Si-supplemented group over the 24 h post-dose period following ingestion of the orthosilicic acid solution (i.e. over and above baseline urinary Si excretion) was 16.5 ± 2.7 mg (range 14.4 – 20.5 mg) and this accounted for 57.0 ± 9.5% (range 49.8 – 71.0%) of the supplemental Si dose (28.9 mg) ingested.

### Faecal Si excretion

**Figure 3:**
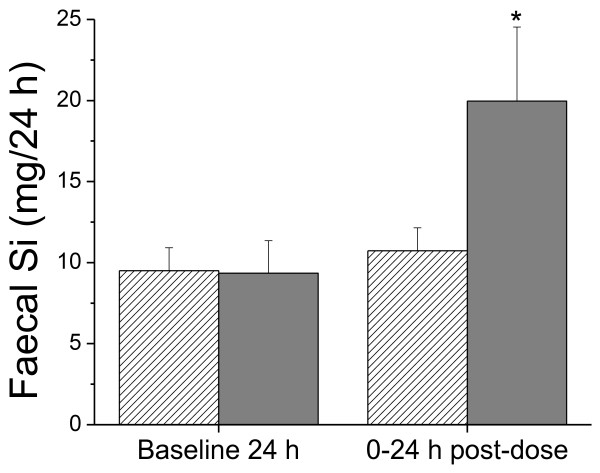
**Faecal silicon excretion.** Mean (± SD) faecal excretion of Si following ingestion of UHP water (*stripped bars*) and UHP water containing 28.9 mg Si (*grey bars*) in six subjects. *P = 0.002 (Mann–Whitney Rank test).

Mean total baseline 24 h faecal excretion of Si was 9.5 ± 1.4 (range 8.0 – 11.5) and 9.4 ± 2.0 (range 6.7 – 11.5) mg, respectively, in the control and Si-supplemented groups (Figure 3) and did not change markedly in the control group following ingestion of UHP water. However, the ingestion of the orthosilicic acid solution led to a significant increase in faecal Si content in the 24 h post-dose collection compared to baseline (*P* = 0.002; Figure 3). This increase in faecal Si excretion in the Si-supplemented group, by + 11.4 ± 2.7 mg (range + 7.6 – 14.9 mg) over 0–24 h period, accounted for 39.3 ± 9.4% (range: 26.4 – 51.6%) of the ingested supplemental Si dose (28.9 mg).

### Silicon balance

In the Control group urinary and faecal Si excretion over the 24 h post-dose period accounted for 99.6 ± 8.1% (range 90.6 – 112.9%) of the total Si intake over that period, whilst in the Si-supplemented group average recovery from urine and faeces was slightly less at 94.8 ± 9.4% (range 83.3 – 105.6%; Table 3). Of the supplemental Si dose ingested (28.89 mg), 27.83 ± 1.67 (range 25.6 – 29.5) mg (or 96.3 ± 5.8%; range 88.5 – 102.2%) was recovered from urinary and faecal excretion over the 24 h post-dose period in the Si-supplemented group.

**Table 3:** Silicon intake, excretion, and balance over 24 h period (study day 1)

	**Control group (n = 6)**		**Si-supplemented group (n = 6)**	
	**Mean ± SD**	**Range**	**Mean ± SD**	**Range**
Silicon intake				
Si supplement (mg)	-		28.89	
Dietary Si intake (mg)	23.86		23.86	
Total Si intake (mg/24 h)	23.86		52.75	
Silicon excretion				
Urinary Si (mg/24 h)	13.34 ± 2.48	(11.36–18.21)	29.41 ± 3.55	(25.44–35.78)
Faecal Si (mg/24 h)	10.74 ± 1.41	(8.74–12.02)	19.96 ± 4.58	(14.31–25.53)
Total excretion (mg/24 h)	23.77 ± 1.94	(21.62–26.95)	50.02 ± 4.98	(43.93–55.68)
Silicon balance (mg)	0.09 ± 1.94	(-3.09–2.24)	2.73 ± 4.98	(-2.93–8.82)

## Discussion

The present study investigated the balance in excretion of silicon *versus* its intake, using a single dose of typical dietary silicon (28.9 mg) in healthy human volunteers on a standardized diet. Our results show that, within error, all (96 ± 6%) of the ingested dose was excreted in urine and faeces over the 24 h post-dose period. Whether it is, fully, the same Si being excreted that is being absorbed will need to be addressed with a different study design. However, to get within 5% of intake with variance of ~ 6% is better than may have been expected, especially with the complexities of faecal and urine collection and analysis [17,22]. Typical recovery from such studies, even with radiolabelled compounds, can be 80% or less, much less than from animal studies [23,24]. The high renal clearance of Si and the lack of interaction with serum proteins probably aids recovery [25,26].

As mentioned previously, 100% recovery is expected if (a) Si metabolism is regulated but the subjects are in Si balance (i.e. are Si replete) or (b) if Si has no active biological function and thus Si metabolism is not regulated at all. This study cannot prove which is true but we believe that the former is more likely based on previous murine data where urinary Si output was found to be conserved in Si-deprived animals to maintain tissue Si levels [15]. To now show this in humans we will need to repeat the study with subjects who are in negative Si balance (i.e. Si deplete at the start of the supplementation period by prior dietary Si deprivation for a week or so). Supplementation with the Si dose should then result in less Si being excreted, as more is retained to replenish the depleted body Si pool, compared to a Si-replete group.

Secondly, from this current work, we cannot be certain that the Si excreted in urine and faeces all originated from the ingested Si dose and that there was not some exchange with the body Si pool- as for example occurs with dietary phosphate [27]. This can only be answered with an isotope label study, where isotopic Si is used for the dose solution to discriminate it from Si of the body pool and from dietary sources (i.e. the meals ingested). However this is also not straight forward. ^31^Si and ^32^Si are both radioactive and would result in exposure to radiation (beta decay) with short (t½ = 157 min) or long (t½ = 153 years) half-lives, respectively. Using a stable isotope such as ^29^Si would avoid radioactive exposure but it has a high natural abundance (ca. 5% of all endogenous Si(OH)_4_). Hence, a relatively accurate balance, as now proven is possible in this study, will be key to the success of the follow on stable isotope work. Moreover, with recent developments in inductively coupled plasma – mass spectrometry methods, to measure ^28^Si and ^29^Si in biological samples [28], we are confident that it will now be possible to discriminate the source of excreted Si (i.e. all ‘washed through’ following ingestion or some from the body pool following exchange with absorbed silicon). Both this question and that of Si retention following oral Si challenge to Si-depleted volunteers are big questions in human Si metabolism and this study proves that they may now be answered with carefully designed isotopic balance studies in Si replete and deplete individuals. In the work presented herein subjects were carefully matched to reduce variability between the two groups, however a cross-over design is undoubtedly more robust to really minimise inter-individual variation in silicon handling [29,30]. Thus, although more burdensome to the subjects, for the future work a cross-over study design will be seriously considered.

Finally, measurement of faecal Si excretion for the first time in a human study, as we report here, allowed the absolute absorption of Si from the orthosilicic acid dose solution to be estimated which, at 61 ± 9% of the ingested dose, is similar to the estimate from total urinary Si output over the 24 h collection period (57 ± 10%). These estimates are comparable with previous data (absorption being stated as ~ 50–60% of the ingested dose) from shorter urine collections, 0–6 or 0–8 h post-dose [20,25,29-34]. Hence, we can conclude that (a) urinary silicon does measure silicon absorption following oral Si challenge and (b) in general 0–6 or 0–8 h urinary collections are adequate to estimate absorption/bioavailability of Si from readily absorbed dietary sources, supplements and test solutions (Si materials requiring prolonged digestion prior to absorption may differ in this respect as previously noted [30].

## Conclusions

In conclusion the present study reports that urinary and faecal Si excretion can be measured with high precision (with inter-subject variance ~ 6%) and that in normal healthy subjects who are presumed to be in Si balance, ingestion of a soluble dose of Si results in an equivalent quantity being excreted within 24 h. We confirm that urinary silicon may be used as an accurate measure of silicon absorption, assuming robust study design. This work also provides clear evidence that a proper isotope-based balance study can now be undertaken in silicon replete and depleted volunteers to inform on homeostasis of silicon and thus provide strong evidence (or not) for beneficial utilisation of the element by humans.

## Abbreviations

AUC: Area under the (serum) curve; HCl: Hydrochloric acid; HNO3: Nitric acid; ICP-OES: Inductively coupled plasma-optical emission spectrometry; OSA: Orthosilicic acid; SD: Standard deviation; Si: Silicon; Si(OH): Orthosilicic acid; UHP: Ultra-high purity.

## Competing interests

All authors declare that they have no competing interests.

## Authors’ contributions

The authors’ contributions were as follows: SSP, AS and RJ designed the research; SS conducted the research; RJ and AS had study oversight; SSP and RJ analysed the data; SSP, JJP and RJ wrote the paper & had primary responsibility for final content. All authors read and approved the final manuscript.
